# Changes in Orientation Behavior due to Extended High-Frequency (5 to 10 kHz) Spatial Cues

**DOI:** 10.1097/AUD.0000000000001113

**Published:** 2021-08-09

**Authors:** William M. Whitmer, David McShefferty, Suzanne C. Levy, Graham Naylor, Brent Edwards

**Affiliations:** 1 Hearing Sciences – Scottish Section, University of Nottingham, Glasgow, United Kingdom; 2 Earlens Corporation, Menlo Park, California, USA; 3 Institute of Health and Wellbeing, University of Glasgow, Glasgow, United Kingdom

**Keywords:** Extended bandwidth, Hearing aids, Orientation behavior, Spatial hearing

## Abstract

**Objectives:**

Current hearing aids have a limited bandwidth, which limits the intelligibility and quality of their output, and inhibits their uptake. Recent advances in signal processing, as well as novel methods of transduction, allow for a greater useable frequency range. Previous studies have shown a benefit for this extended bandwidth in consonant recognition, talker-sex identification, and separating sound sources. To explore whether there would be any direct spatial benefits to extending bandwidth, we used a dynamic localization method in a realistic situation.

**Design:**

Twenty-eight adult participants with minimal hearing loss reoriented themselves as quickly and accurately as comfortable to a new, off-axis near-field talker continuing a story in a background of far-field talkers of the same overall level in a simulated large room with common building materials. All stimuli were low-pass filtered at either 5 or 10 kHz on each trial. To further simulate current hearing aids, participants wore microphones above the pinnae and insert earphones adjusted to provide a linear, zero-gain response.

**Results:**

Each individual trajectory was recorded with infra-red motion-tracking and analyzed for accuracy, duration, start time, peak velocity, peak velocity time, complexity, reversals, and misorientations. Results across listeners showed a significant increase in peak velocity and significant decrease in start and peak velocity time with greater (10 kHz) bandwidth.

**Conclusions:**

These earlier, swifter orientations demonstrate spatial benefits beyond static localization accuracy in plausible conditions; extended bandwidth without pinna cues provided more salient cues in a realistic mixture of talkers.

## Introduction

Hearing prostheses historically have had a limited bandwidth of amplification in the extended high frequency region above 5 kHz due to multiple aspects of their design, from lower sampling rates to the acoustic tubing used in many models of hearing aids to the power required to provide audible output and gain to achieve audibility for users who have more than mild high frequency hearing loss. The current useable frequency range of conventional acoustic hearing aids is approximately 0.25 to 6 kHz, though the useable upper frequency limit can vary from 3.5 to 8 kHz (Kimlinger et al. 2015). Recent advances in signal processing as well as novel methods of transduction (e.g., direct non-acoustic actuation of the tympanic membrane) have demonstrated a greater useable frequency range, with useful audible amplification achieved through 10 kHz for those with up to severe high frequency hearing loss (Gantz et al. 2017; Arbogast et al. 2018).

There have been mixed conclusions regarding the benefit of extended high frequency amplification in the literature investigating aspects of subjective preference and speech understanding. For those with residual hearing above 6 kHz, qualitative preference for extending the bandwidth from 5 to 5.5 to 8 to 11 kHz has been demonstrated in listeners with hearing impairment (Ricketts et al. 2008; Brennan et al. 2014; Van Eeckhoutte et al. 2020). With regards to speech, several studies have shown consonant recognition benefits for increasing bandwidth, both in quiet (Turner & Henry, 2002; Simpson et al. 2005) and in noise (Horwitz et al. 2008; Füllgrabe et al. 2010). Further, Donai and Halbritter (2017) has shown talker-sex identification benefits for speech high-pass filtered above 5 kHz. Amos and Humes (2007), however, found no difference in word recognition in mild noise [+5 and +20 dB signal-to-noise ratio (SNR)] for elderly hearing-impaired listeners when the upper frequency limit was extended from 3.2 to 6.4 kHz. Silberer et al. (2015) also found limited improvement in speech recognition with extended bandwidth when congruent visual information was available for normal-hearing listeners.

Spatial advantages resulting from extended high frequencies is another area of interest due to increased access to short-and long-term interaural level difference cues and spectral shaping caused by the pinnae. Numerous recent studies have indicated potential spatial advantages of extending the bandwidth beyond 5 kHz (Levy et al. 2015; Donai and Schwartz 2016; Jakien et al. 2017). Levy et al. (2015) showed improvements in speech understanding with increasing bandwidth from 4 to 10 kHz for both normal-hearing and hearing-impaired listeners when simulated target and masker locations were asymmetrically separated, and improvements for normal-hearing listeners when the noises were diffusely presented, but not for the hearing-impaired listeners. Jakien et al. (2017) also found a spatial-separation benefit when targets and maskers were presented at effective bandwidths of 5 versus 3 kHz. Spatial advantage has been proposed to be in the spectral signatures in the higher frequencies for particular locations caused by the shape of the pinna (e.g., Best et al. 2005; Brungart et al. 2017). However, with the most common hearing aid design, the behind-the-ear (BTE) model, the microphone is just above the pinna, hence many of these spectral details are lost. Even with this loss of directionally dependent high frequency filtering, the high frequency portion of a signal still contains interaural level differences due simply to the shadowing of sound by the head. Further, these differences generally increase with increasing frequency. By increasing the upper frequency limit of a BTE hearing device, these interaural level differences should become more prominent and thus potentially more accessible to the wearer. In reverberant environments where the low-frequency ITD cues are less reliable due to reflections, high-frequency ILDs and envelope ITDs can dominate (e.g., Kerber & Seeber, 2013). It is therefore possible that extending bandwidth, even with a BTE microphone location, could provide salient spatial cues which may be beneficial to users to locate as well as segregate signals of interest.

To explore the question of whether there would be any spatial benefits to extending bandwidth in the absence of pinna cues, we used a method developed by Brimijoin et al. (2010, 2012, 2014) to probe differences in dynamic localization behavior, especially where localization accuracy itself may not be affected. Individuals with audiometrically normal hearing to the high frequencies and symmetric hearing (see Participants section in Materials and Methods for definitions) were tested in a condition where access to pinna cues were removed. Localization accuracy, particularly in terms of lateral judgements, has been shown to be only modestly affected by differences in hearing aids (Akeroyd and Whitmer, 2016). However, dynamic localization measures, such as the detection of angular or radial movement (Lundbeck et al. 2017) and orientation measures, such as duration to reorient to a source (Brimijoin et al. 2014), have been shown to be more sensitive to changes in hearing aid technology. Brimijoin et al. (2014) found differences in behavior with and without directional microphones by probing difficult SNRs, where detection of the signal could require head movement. In this study, we used a more favorable (and more realistic; cf. Smeds et al. 2015) SNR. Hence, the current study focuses on the spatial benefits of extended bandwidth in segregating an auditory scene when all stimuli are detectable.

The hypotheses for this experiment are that the additional bandwidth will make the cues for locating the target more salient, which will (1) increase lateral certainty (i.e., reduce misorientations), (2) decrease reaction time (i.e., decrease the delay before reorienting), and (3) increase orientation speed (i.e., peak velocity). Female and male targets were used to probe differential benefits which may be revealed with access to the extended high frequencies. What benefits would be expected to come from increased saliency? A quick, smooth orientation to target minimizes loss in the signal due to reorientation (Brimijoin et al. 2014), while also maximizing the available congruent visual cues. Optimal orientations would also minimize the inevitable interaural distortions due to adaptive signal processing (e.g., automatic gain control) that occur while the head is in motion (e.g., Brimijoin et al. 2016). While there are well documented benefits for the spatial separation of targets from their distractors, even in distance (Westermann and Buchholz 2017), there remains a lack of documented correlation between localization ability and the ability to use spatial cues (Noble et al. 1997). As a behavior dependent on those spatial cues, orientation to a sound source in an ecologically viable environment provides insight into the most direct use of those cues, bridging the gap between self-motion and spatial benefit (cf. Grimm et al. 2020).

## Materials and Methods

### Participants

**Fig. 1:**
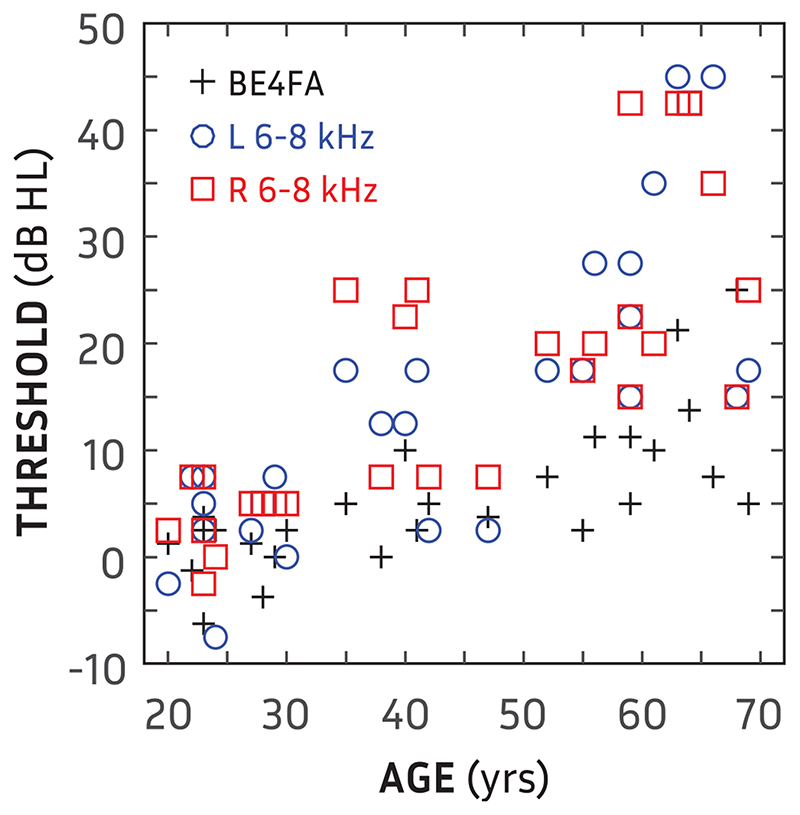
High-frequency (6 and 8 kHz) pure-tone threshold averages for left (blue circles) and right (red circles) ears as a function of age for all participants. Crosses show better-ear four-frequency pure-tone average (BE4FA) as a function of age.

This study was approved by the West of Scotland research ethics service (WoS REC(4) 09/S0704/12). Written informed consent was obtained from all participants before commencing experimentation. Participants were recruited from local hearing clinics, the student population of the University of Glasgow and employees of the Scottish section of the Institute of Hearing Research. Pure-tone thresholds were obtained using the modified Hughson-Westlake method (British Society of Audiology, 1981). In total, 34 participants were recruited (16 female). However, six were excluded due to either a high-frequency pure-tone average between 6 and 8kHz >45 dB HL (hearing level) or an interaural asymmetry >15 dB HL over the same frequency range. As shown in Figure 1, high-frequency pure-tone average, averaged across ears, was significantly correlated with age (*r* = 0.80; *p* « 0.001), as was better-ear four-frequency pure-tone average (BE4FA; *r* = 0.74; *p* « 0.001).

### Apparatus

Participants sat in a freely rotating chair in the center of an azimuthal circular array of 24 loudspeakers (3.5 meter diameter, 15° separation) located in a large sound-proof audiometric booth (4.3 × 4.7 × 2.9 m). The ceiling and walls were covered with acoustic foam to reduce reflections and a thin black cloth—tested to be acoustically transparent - was draped over the loudspeakers to avoid visual anchors.

**Fig. 2:**
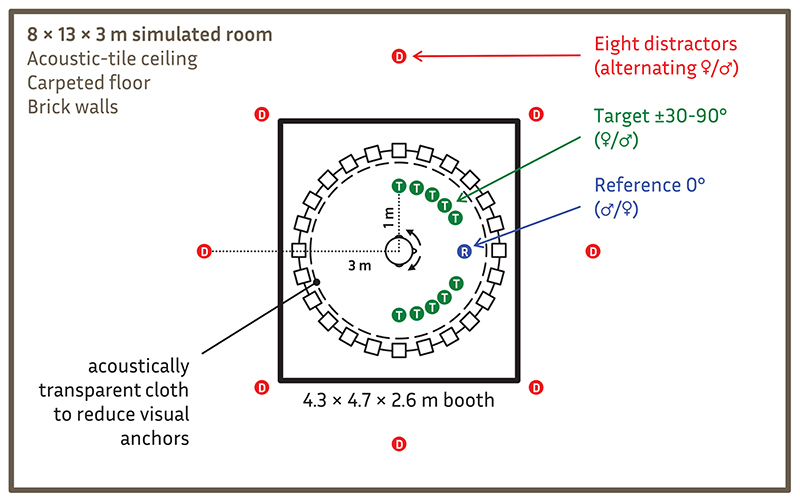
Schematic of possible simulated target (green T), reference (blue R), and eight distractor (red D) sources in a simulated reverberant 8 × 13 × 3 m space (gray) presented through a 24-loudspeaker array in a sound-dampened 4.3 × 4.7 × 2.6 m chamber (black).

An 8 × 13 × 3 m room (see Figure 2) was simulated using ODEON Room Acoustics Software (v11, www.odeon.dk). The simulated room had a 6-mm pile carpet floor, an acoustic-tiled ceiling, and smooth brickwork on the walls with a reverberation time (*T*_60_) varying across frequency from 1 sec at 125 Hz to 0.4 sec at 8 kHz. Twenty-four channel impulse responses derived from a nearest-loudspeaker auralization (Favrot & Buchholz 2010) were created for each target and distractor location. The spatial configuration of the simulation was designed to create (a) a realistic, favorable SNR with all sources speaking at nearly the same level, and (b) relatively punctate (interaural coherence = 0.89) near-field targets within the critical distance (1.2 m using Sabine’s estimation) that were verbally confirmed in practice runs to be easy to detect in the presence of diffuse far-field distractors (interaural coherence = 0.28).

Stimuli were routed from a PC running Matlab and equipped with a multi-channel sound card (RME HDSPe MADI) at a sample rate of 44.1 kHz to a pair of AD/DA converters (Ferrofish A16-MKII). The analog output was amplified via 6 four-channel amplifiers (ART SLA4) before being presented over the loudspeakers (Tannoy VX6), calibrated to equal sound pressure level ±0.1 dB at 1 kHz. Participants’ head movements were tracked via a head-worn “crown” of infrared reflectors illuminated and recorded at a sample rate of 100 Hz by 6 wall mounted cameras (Vicon MX3+). The cameras were linked to a Vicon MX Ultranet unit. The motion capture data from the Ultranet was returned to Matlab on the same PC via the Vicon Tracker software package and the Vicon DataStream SDK.

To emulate the BTE microphone position, omnidirectional microphones (Sound Professionals SP-TFB-2) were fixated above both pinnae at the front BTE microphone position by attaching the teardrop-shaped microphone housings into the grooves of plastic ear hooks that went around the rear of the pinna. The lateral orientation of the microphones was verified using the loudspeaker array to not affect its omnidirectional response in the free-field (directivity index 0.95 to 1.02 from 0.125 to 16 kHz). The ear hooks were held in place, if necessary, with a small piece of microporous tape attached to the rear of the pinna. The microphone signals were amplified (Zoom H4N recorder as pre-amp routed to an iBasso Mamba headphone amplifier) and presented via earphones (Etymotic Research ER-2; attenuation increased from approximately 30 dB at 0.1 to 1 kHz to approximately 40 dB at 4+ kHz). This provided a zero-latency, near-flat (±5 dB from 0.25 to 8 kHz), near-linear (1.00 to 1.04:1 from 45 to 95 dB SPL at 1 to 4 kHz) response as measured using an IEC-711 ear simulator in a test box (Interacoustics TBS25).

### Stimuli

Speech stimuli were taken from an uninterrupted speech test developed by MacPherson and Akeroyd (2013), originally recorded at 44.1-kHz sample rate (i.e., with signal energy well above 10 kHz). These signals consisted of two talkers, one male and one female, reading the same Sherlock Holmes story. Each recording was segmented into >5 second lengths at the same pauses, so that when the signal switched from the reference to target position, the target talker was finishing the reference talker’s statement. Two other talkers, one male and one female, were used as eight distractor noises in alternating distractor locations (e.g., female at 0°, male at 45°, etc.) to create a background babble. To avoid confusion arising from duplicate presentation of the same material, the speech samples used as distractor noises were from a different section of the story as those used for signals. Stimuli were convolved with the appropriate impulse responses before presentation.

On an equal but random number of trials, the reference signal was the male or female talker, which was presented from the simulated 0° near-field position for 1 seconds. This origin (0°) was based on the participant’s head position, hence interpolated from the nearest actual loudspeakers; that is, the simulated room was re-oriented on every trial to match the position of the participant’s head. The target signal was the opposite sex talker presented 1 s after the reference for 4 s from one of 10 simulated locations. Simulated reference and target sources were located 1 m from the head position, with the target at one of the ten locations (±30, 45, 60, 75, or 90° relative to the participant’s head). Simulated distractor sources were located 3 m from the listeners head position at angles of 0, 180, and ±45, 90, and 135°. A 100-ms linear offset gate was applied to both the reference and target signal to eliminate transients.

Reference, target, and distractor stimuli were low-pass filtered (8th order Butterworth) on each trial with either a 5- or 10-kHz cutoff frequency. The reference and target presentation levels were calibrated to be a long-term average of 66 dB A for both low-pass conditions, measured from the center of the loudspeaker array using a sound level meter (B&K 2260 Observer with B&K 4189 ½ inch microphone). The overall presentation level of the eight simultaneous distractors was also calibrated to be 66 dB A at the center of the array. The resulting long-term SNR on every trial was 0 dB.

### Procedure

First, audiometric data were collected for each participant. The motion tracking equipment was then calibrated to each individual’s ear-nose plane. For this calibration step, as well as the head-worn crown, participants temporarily wore eyeglasses with a reflective marker at the bridge of the nose and two wiremounted reflective markers which could be adjusted to sit at the entrance to the ear canals. Corrective alignments were made using the Vicon Tracker software. After motion-tracking calibration the eyeglasses were removed.

The participants then had the BTE microphones fitted and were instructed how to fit the foam-tipped earphones themselves. Verbal confirmation was sought that occlusion had occurred. If necessary, different sized foam ear plugs were used if occlusion was incomplete or discomfort was evident. All equipment wiring was routed around the participants head and shoulders, as to not constrict participant movement, to amplifiers mounted on the back of the chair. The amplifiers were then powered on and again verbal confirmation was sought, this time that each BTE microphone was working correctly. During stimuli presentation, participants were allowed and encouraged to make natural head movements or spin in the chair to help them localize but were explicitly asked not to move the chair from the central position. All complied.

Participants were first given instructions. They were to imagine they are at a busy party with several friends nearby telling a story in turn. On each trial they would hear a voice from ahead for 1 sec, and then a voice of the opposite sex in another location continuing to tell the story. They should turn their head and/or chair as quickly and accurately as is comfortable so that they are directly facing the new talker and hold that position for a few seconds till the next trial starts. Five randomly chosen practice trials were run. There were four repeats of both low-pass cutoff (5 and 10 kHz) conditions for both male and female-talker targets at all 10 possible locations resulting in 160 trials, presented in randomized order, which took each participant approximately 40 minutes to complete with a short break after every 40 trials.

## Results

On every 5-sec trial, each participant’s orientation behavior was recorded as the instantaneous head yaw angle recorded every 10 msec. Some orientations were discarded due to sudden movement at the start of a trial or tracking error (e.g., signal dropout); 2 to 6 orientations (1 to 4%) per participant were discarded. For each orientation, the first 1 sec—during reference presentation—was not included in the analysis. That is, the onset of the target stimulus 1 sec into the trial is considered time zero for all measures, and the overall orientation duration was 4 sec.

### Analysis

**Fig. 3:**
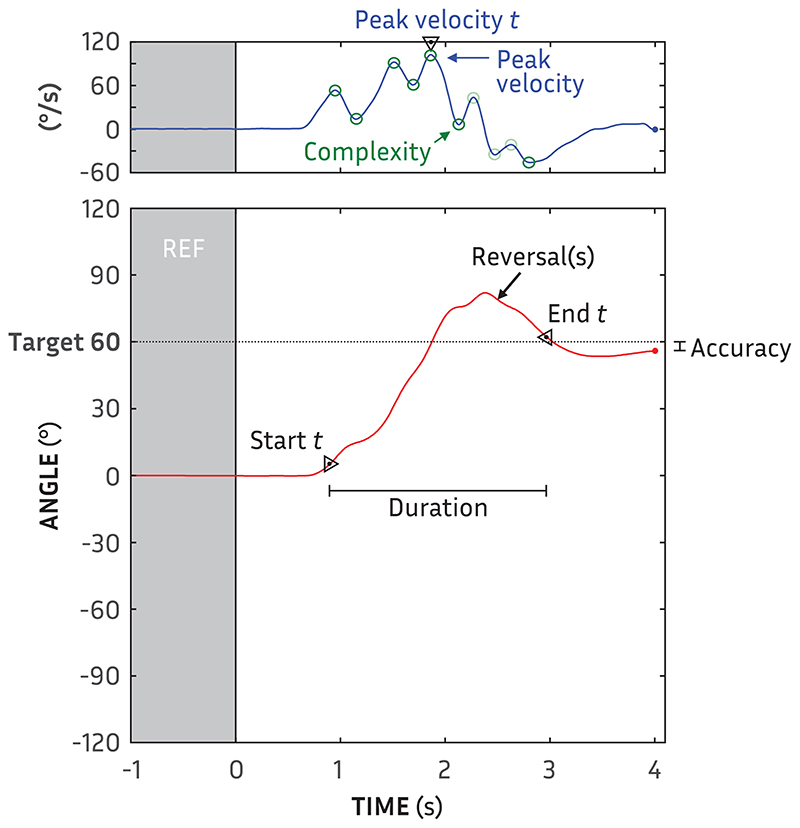
Example of trajectory angle (red line) as a function of time showing the all orientation measures: start time, duration, end time, reversals, misorientations, peak velocity, peak velocity time and complexity of the trajectory, as well as accuracy.

The following aspects of each orientation were quantified: trajectory start, trajectory end, trajectory duration, accuracy, peak velocity, time of peak velocity, complexity, rate of misorientations, and rate of reversals (please see example in Figure 3). While non-exhaustive, we have chosen a set of measures that are both descriptive of orienting behavior (Brimijoin et al. 2014) and sensitive to changes in behaviors (Maurer et al. 2018), including orientation accuracy for comparison purposes. The previously unreported interdependence and redundancy of these measures is considered later in the Results. Natural unfixed head movements can vary by as much as 5° (König and Sussmann 1955). We have therefore used a 5° threshold to determine variations in the orientation.

Accuracy, the traditional measure of localization ability, was calculated as the absolute difference between the target angle and head angle at the end of orientation. The trajectory start time (s) of every orientation was determined from the first point where the trajectory was ±5° from its position at orientation start. The start time measure provides a spatial hearing analogy to reaction time, indicating the saliency of the decision which way to turn (cf. Maurer et al. 2018). The trajectory end time (s) was determined from the last point that the trajectory was ±5° from its position at orientation end. The trajectory duration (s) was trajectory start time subtracted from trajectory end time; it provides insight into how much of a speech utterance could be lost in re-orientation (Brimijoin et al. 2014). Reversal rate, a potential indicator of saliency during movement, was calculated from the average number of times over the course of the trajectory that its direction changed (i.e., zero-crossover points in the velocity function) by more than 5°. Misorientation rate, another potential indicator of lateral saliency, was the number of initial movements, averaged across repetitions of each angle × stimulus condition, which were toward the opposite side as the target.

Trajectory velocity (°/s) was determined from the derivative of movement (i.e., Δθ/Δ*t*). The value and time of peak trajectory velocity—the absolute maximum of trajectory velocity—have been previously shown to be key indicators of changes in movement behavior (e.g., Hore et al. 1995; Smeets et al. 2002; Maurer et al. 2018). A simple trajectory should have a smooth motion with minimal deviations or hesitations during movement. While a smooth rapid movement will ideally be ballistic (Leung et al. 2008; Miller et al. 2020), not all smooth trajectories can be described with a quadratic function (cf. Brimijoin et al. 2014; Whitmer et al. 2020). All hesitations, though, would be manifest as inflection points in the trajectory velocity. We therefore calculated complexity as the rate of inflections points (per second): the number of zero-crossover points in the trajectory acceleration (versus velocity for reversals) divided by the trajectory duration. To reduce the influence of small oscillations in velocity, trajectories were smoothed with a 100-ms Hann window, and successive inflection points that were <5° from the preceding inflection were excluded.

## Main Results

**Fig. 4:**
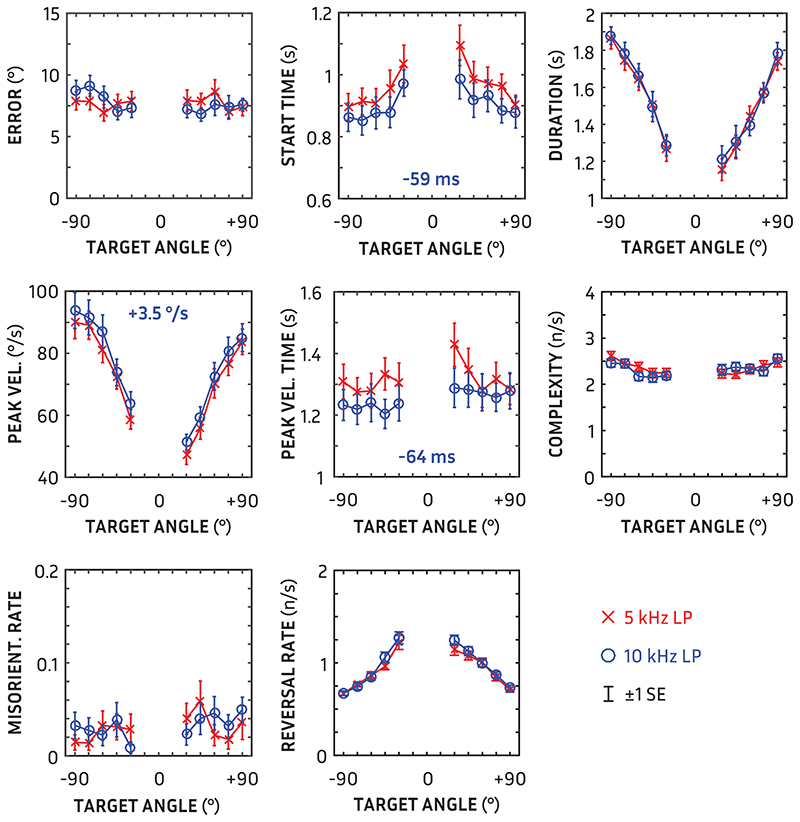
Orientation measurements in separate panels for 5-kHz low-pass (red crosses) and 10-kHz low-pass (blue circles) stimuli as a function of target angle. Error bars show ±1 standard error. Mean differences for measures with statistically significant differences are shown (in blue) in the appropriate panel (all *p* « 0.001).

Mean orientation measures as a function of target angle for the three stimulus conditions (5-kHz LP, 10-kHz LP, and 5 to 10 kHz BP) are shown in Figure 4. There was no statistically significant effect of target-voice sex for any measure (all *p* ≫ 0.05); hence, all results are collapsed across target sex, resulting in eight repeats per angle and low-pass condition. Non-integer degrees of freedom in reported repeated-measures analyses of variance are based on the Greenhouse-Geisser correction (∈) for non-sphericity.

### Accuracy (Error)

There was no significant difference in accuracy between 5 and 10 kHz stimuli [*F*(1, 27) = 0.00; *p* = 0.98]. Localization error was not affected by target angle [*F*(4.8, 129.9) = 1.31; *p* = 0.23]. The overall mean error was 7.7°. To check for potential orientation biases, participants’ signed error was also calculated; the overall mean signed error (−1.2°) was not significantly different from zero [*t*(27) = −1.16; *p* = 0.26]. Individual orientation bias ranged from −14.1 to +9.7°.

### Start Time

The delay between the onsets of the target and participants’ movement was affected by stimulus bandwidth [*F*(1, 27) = 38.25; *p* « 0.001; η^2^ = 0.59]. Participants began their movements toward the target 59 ms earlier when presented with 10-kHz than 5-kHz low-pass stimuli. Trajectory start time was also affected by target angle [*F*(4.9, 132.8) = 9.06; *p* « 0.001; η^2^ = 0.21]; post hoc comparisons revealed that movements toward the nearest targets at ±30° were delayed 110 ms more on average than farther targets. The delay was also asymmetric [*F*(1,27) = 9.27; *p* = 0.005; η^2^ = 0.26]; on average, participants moved 36 msec earlier toward targets to the left than the right.

### Duration

There was no significant difference in trajectory duration between 5 and 10 kHz low-pass stimuli (*F*(1,27) = 0.30; *p* = 0.59; *p* = 0.59). Trajectory duration was affected by target angle as expected [*F*(4.5, 124.5) = 42.40; *p* « 0.001; η^2^ = 0.61]; it took longer to orient to farther away—147 msec per 15° on average. There was also an asymmetry in the target-angle effect: turns to the right were on average 167 msec shorter than turns to the left [*F*(1,27) = 17.09; *p* < 0.001].

### Peak Velocity

Peak velocity during participants’ trajectory was affected by stimulus bandwidth [*F*(1,27) = 25.66; *p* « 0.001; η^2^ = 0.49]; velocity increased with increasing bandwidth by 3 °/s. Peak velocity was also affected by target angle [*F*(3.2, 86.8) = 73.65; *p* « 0.001; η^2^ = 0.73]: the greater the target angle, the greater the peak velocity achieved. Peak velocities were also generally asymmetrical [*F*(1,27) = 31.58; *p* « 0.001; η^2^ = 0.54]: movements to the left were faster by 10 °/s on average than movements to the right.

### Peak Velocity Time

The peak velocity time—the point in the trajectory when the peak velocity occurred—was affected by stimulus bandwidth [*F*(1, 27) = 17.78; *p* < 0.001; η^2^ = 0.40]. Participants reached maximum velocity 64 ms earlier for 10-kHz than 5-kHz low-pass targets. Peak velocity time was not affected, however, by angle [*F*(4.71, 127.36) = 1.90; *p* = 0.10].

### Complexity

The complexity of trajectories, measured as the rate of fluctuations in trajectory velocity, was unaffected by stimulus bandwidth [*F*(1, 27) = 0.77; *p* = 0.39]. Complexity was affected by target angle [*F*(7.22, 41.82) = 4.66; *p* « 0.001; η^2^ = 0.15]; complexity modestly increased with increasing target angle without any significant symmetrical differences [*F*(1,27) = 0.13; *p* = 0.72].

### Reversals

The number of reversals in direction per second was unaffected by stimulus bandwidth [*F*(1,27) = 3.22; *p* = 0.08]. There was an effect of target angle on reversal rate [*F*(2.92, 78.98) = 38.98; *p* « 0.001; η^2^ = 0.60]; the number of reversals increased with decreasing target angle without any significant symmetrical differences [*F*(1,27) = 3.36; *p* = 0.078].

### Misorientations

The rate of misorientations at the outset of movement was not affected by stimulus bandwidth [*F*(1,27) = 0.15; *p* = 0.70]. Misorientation rate was also unaffected by target angle [*F*(4.37, 117.97) = 1.31; *p* = 0.27]. Participants initially turned in the wrong direction on an average of 3.1% of trials.

### Correlations and Data Reduction

When controlling for the correlations of age with BEA (*r* = 0.74; *p* « 0.001) and age with HFA (*r* = 0.80; *p* « 0.001), there was no correlation between age, BEA or HFA and any of the orientation measures.

To reduce the number of measures necessary to report in orientation behavior, we looked for potential redundancies in the measures using a principal components analysis, averaged across conditions and angles, using varimax rotation. While the sample size is relatively low for a principal component analysis, as was its Kaiser-Meyer-Olkin measure of sampling adequacy (0.45; Kaiser and Rice, 1974), the communalities were relatively high (0.56 to 0.91) as were most component loadings (see Table), indicating that the analysis is feasible (MacCallum et al. 1999). Three components accounted for 76% of the variance as shown in Table. The first component consisted of start time, reversals, peak velocity time and peak velocity. Start time strongly correlated positively with peak velocity time, indicating that individuals who started slower reached their maximum orientation speed later, and correlated negatively with reversals, indicating that those who started earlier were more prone to reverse their direction (e.g., overshoot the target). The second component consisted of duration, complexity and misorientations. Duration was positively correlated with misorientations, indicating those who took longer were more prone to start in the wrong direction (nb. there were very few misorientations) but negatively correlated with complexity, indicating the rate of hesitations (complexity) decreased with longer-duration trajectories. The third component was just error.

## Discussion

In a sample of normal-hearing users, there was useful spatial information between 5 and 10 kHz even without pinna cues. Participants oriented earlier and more swiftly towards a target in a realistic scenario when the acoustic scene was low-pass filtered at 10 versus 5 kHz. The evidence for a universal behavioral impact of extra bandwidth, however, was not complete. A more spatially salient target could have been expected to also lead to quicker trajectories (i.e., shorter durations), fewer misorientations at the start of the trajectory and less complex movements towards the target; there were no differences in these measures. In our previous orientation study (Brimijoin et al. 2014), these three measures—duration, misorientation, and complexity—all showed significant differences between directional microphone modes. The previous complexity measure was the RMS difference to a logistic fit of the trajectory; using that previous metric, or a minimum polynomial fit (Brimijoin et al. 2010; Whitmer et al. 2020), with the current data also did not show any difference due to bandwidth. The stimuli in Brimijoin et al. (2014) were presented at SNRs individually tailored to be difficult, and were also presented at locations >90° from midline. Given that the present experiment used a higher SNR and eliminated pinna cues in the 5 to 10 kHz region, it is perhaps not surprising that the evidence of spatial saliency was limited to onset and velocity measures. Of note, studies of movement have highlighted onset and peak velocity as kinematic indicators or “landmarks” of behavioral change (Smeets et al. 2002; Maurer et al. 2018). That is, the timing of the start of one’s movement, the peak velocity, and its timing can distinguish one intentional movement from another.

With extended bandwidth to 10 kHz, participants began their trajectories 59 msec earlier and reached peak velocity 64 msec earlier. These are relatively small differences compared with the average delay in movement and movement duration (933 and 1530 msec, respectively). Nevertheless, the results are comparable to previous findings (e.g., Oude Nijhuis et al. 2007; Pals et al. 2015) if we consider these orientation onset time measures to be a proxy for auditory-localization reaction time. The key finding in the highly cited study of listening effort by Sarampalis et al. (2009) was that reaction time was reduced by 51 ms re an average reaction time of 714 ms when hearing aid noise reduction was active, albeit only in one, low SNR condition. More broadly, the effect of incrementally increasing complexity on visual search reaction times is 20 to 40 ms (Wolfe, 1998). Hence, the small effects observed here are within the expected scale of results for changes in behavior. Further, this behavior was measured during presentation in each condition, and not the reaction time from silence, so would not be susceptible to threshold effects (cf. Piéron 1914).

### Dependence on Target Azimuth

Many of the orientation measures (start time, duration, peak velocity, complexity, and reversal rates) varied as a function of target angle. Most of this angular dependence appears to be due to the opportunity for increased duration, velocity, and complexity afforded by longer excursions. The modest increase in complexity with angle occurred despite using rate of hesitations to reduce the confound of duration; this may have been due to the larger angles requiring combined trunk/chair-head versus head-only movement (cf. Miller et al. 2020), which could have created additional inflections in the trajectory velocity. One dependence of note is the inverse relationship between reversal rate and angle; that is, the number of reversals was greater for smaller target angles. This was most likely due to overshoot—later corrective movements in the trajectory to align with the target signal, combined with the relatively longer available time to make a decision. While peak velocity was heavily influenced by target angle, the peak velocity time was not affected by target angle; that is, the turning speed participants were able to achieve was affected by how far they had to turn, but the point at which they achieved that speed remained, on average, constant. While there were angular dependencies on absolute measures, there was no effect of angle on the differences between 5 and 10-kHz stimuli for any measure.

There were also lateral asymmetries in behavior, namely in trajectory start time, duration and peak velocity, despite the symmetrical arrangement of targets and hearing thresholds of the participants. Previously, it has been assumed that because the error is symmetrical—as it was here—that the orientation behavior is symmetrical, and behaviors were analyzed for angle and not laterality (e.g., Brimijoin et al. 2014). Orientation behaviors are, however, rarely symmetrical, exhibiting both general and individual biases to either the right or left (e.g., van der Kamp & Canal-Bruland, 2011). Here, the orientation asymmetries—earlier and faster but longer-duration trajectories to the left versus right—do not indicate a particular lateral advantage, but rather another orientation bias in need of further exploration.

### Limitations and Future Improvements

The current study used a sample of normal-hearing participants. The relevance of these results to those requiring extended high-frequency amplification for audibility requires further investigation. While measureable differences were found here in ecologically relevant conditions—no pinna cues with all stimuli audible—the scale of those differences may be reduced (i.e., smaller effect sizes) due to the increased variability in hearing-loss pathologies. Furthermore, future investigation should define the ecologically relevant set of conditions for listeners using extended-bandwidth amplification.

Using a scenario employing a nominally valid SNR and continuous speech, we here have shown benefits in orientation for extended bandwidth even without pinna cues. This benefit is potentially conditional on the particular task, and could differ based on the task (e.g., without a reference sound source). It would also be important to know how these benefits might change long-term with usage in real-world listening scenarios. That is, one might expect that a listener would adapt their behavior over multiple sessions, or before and after periods of acclimatization to listening to extended high frequency amplification. This was beyond the scope of the current study which used a randomly interleaved design and listeners who had prior access to high frequency information due to their normal hearing thresholds. In most realistic scenarios, there would also be visual information, which could bolster the ability to orient to a new source, but it is not clear how it would affect an acoustic experimental contrast. Speech signals have a power spectrum that decreases with increasing frequency (i.e., the current target stimuli average power decreased approximately −5 dB/octave from its peak at 500 Hz). This may have limited the effects of extending bandwidth; greater effects might be found with signals having more high-frequency power (e.g., broadband noise), though that would be a different hypothetical use scenario.

We have used orientation measures here to mine spatial benefits from extended bandwidth. In efforts to refine the analysis of orientation, future research should look at the largest contrast possible: unilaterally versus bilaterally aided orientations (Akeroyd & Whitmer, 2016). As an initial refinement of orientation measures, the principal component analysis indicated three dimensions to spatial orientation behavior: (1) certainty, consisting of the control-function measures such as start and peak velocity time (cf. Hore et al. 1995; Maurer et al. 2018), (2) strategy, consisting mainly of duration and complexity, measures that encapsulate the overall behavior, and (3) accuracy. It is of note that accuracy, the predominant measure of spatial benefit, was uncorrelated to the other measures that are more indicative of task behavior. In more difficult tasks and scenarios, detection of the signal itself may be challenging, leading to very different orientation behaviors, which in turn may lead to benefits being expressed in different measures, such as the complexity of the trajectory (cf. Brimijoin et al. 2014). In the current study, the ability to easily detect the stimuli was only established through practice trials, and not measured through a separate detection task. Hence, the extended bandwidth may have also increased detectability (e.g., Monson et al. 2019) as well as spatial certainty. Future research is warranted to explore which measures capture all spatial benefits, but by using a suite of behavioral measures, potential spatial benefits can be more readily captured across different situations.

## Conclusions

These results show there is indeed useful spatial information in the 5 to 10 kHz region without pinna cues that allow for an earlier, swifter orientation towards a target. While accuracy was unaffected, there were changes in orientation behavior that are coincident with the hypothesis that extended bandwidth provides a more salient source location, decreasing reaction time.

## Figures and Tables

**Table:** Results of principal components analysis, showing (unrotated) component loadings (coefficients) for all measures

		Component	
Measure	1	2	3
Start time	−0.88		
Peak vel. time	−0.84		
Peak velocity	0.79		
Reversals	0.62	−0.56	
Duration		0.89	
Misorientation	0.48	0.57	
Complexity	−0.46	−0.57	0.44
Error			0.90
Eigenvalue	3.08	1.95	1.05
Cum. % variance	38%	63%	76%

Measures are grouped by their principal component. For readability, only coefficients considered stable (≥ 0.4; Guadagnoli and Velicer, 1988) are shown. The eigenvalues and cumulative percentage of variance explained are given at the bottom.
